# Time trends and epidemiological patterns of perinatal lamb mortality in Norway

**DOI:** 10.1186/s13028-015-0155-6

**Published:** 2015-09-30

**Authors:** Ingrid Hunter Holmøy, Steinar Waage

**Affiliations:** Faculty of Veterinary Medicine and Biosciences, Department of Production Animal Clinical Sciences, Norwegian University of Life Sciences, P.O. Box 8146 Dep., 0033 Oslo, Norway

**Keywords:** Sheep, Recording system, Time trends, Stillbirth, Neonatal mortality

## Abstract

**Background:**

Perinatal mortality is a major cause of loss in the sheep industry. Our aim was to explore time trends in crude population stillbirth and neonatal mortality rates in Norway. We used data on 6,435,715 lambs from flocks enrolled in the Norwegian Sheep Recording System (NSRS) from 2000 through 2010 for descriptive analysis of trends. Longitudinal patterns of mortality rates were compared for lambs within different levels of variables suspected to be associated with perinatal loss.

**Results:**

There was an approximately linear increase in the annual proportion of stillborn lambs during the study period, from 3.3 % in 2000 to 4.7 % in 2010. In the same time period, average litter size of ewes in NSRS flocks increased from 2.00 to 2.19. However, a steady rise in stillbirth rate was observed within each litter size group, suggesting a gradually increasing impact on stillbirth risk of other, yet unidentified, factors. Average flock size increased during the study period. The highest stillbirth rates were found in the largest and smallest flocks. Early neonatal mortality rates (0–5 days of life) varied from year to year (minimum 2.2 %, maximum 3.2 %) and were invariably higher among triplets and quadruplets than among singletons and twins. Annual fluctuations were parallel within the various litter sizes. A significant overall decreasing trend was present within all litter sizes with the exception of singletons. Weather data for the prime lambing months (April and May) 2000–2010 indicated a relationship between low temperatures and high neonatal mortality rates. At the flock level, there was a significant positive correlation between stillbirths and early neonatal mortality rates (r = 0.13), between stillbirth rates in two consecutive years (r = 0.43) and between early neonatal mortality rates in two consecutive years (r = 0.40).

**Conclusions:**

The substantial increase in ovine stillbirth rate in recent years in Norway was to some extent related to a corresponding increase in the proportion of lambs in triplet or larger litters; however, other factors apparently have contributed. Early neonatal mortality rate exhibited year-to-year variations, partly following temperature fluctuations, which is somewhat unexpected, considering that lambing mainly occurs indoors in Norway.

## Background

Perinatal lamb mortality rates until 7 days of age recorded in studies from various countries range from 9 % to around 20 % [1–4]. These losses have a great impact on profitability in sheep production [5, 6] and are of concern also from an animal welfare perspective [7, 8]. The mortality rates reported were mainly based on studies conducted in single or a few flocks, which limits generalizability of the findings. Quantification of losses in a population requires that reliable mortality records are available from a great number of flocks. Perinatal mortality risk may be influenced by exposures that vary from year to year, and death rates experienced in a single year are not necessarily representative of losses in previous or subsequent years in the population. At the flock level, considerable year-to-year variation in perinatal mortality has been observed, particularly when lambing occurs outdoors and ewes and lambs are exposed to varying weather conditions [9, 10]. Thus, to obtain reliable estimates of perinatal losses in the population, annual mortality rates should be recorded over an extended period of time in a representative sample of flocks. Data recorded over several years could also reveal time trends that deserve closer attention. When combining mortality data with records describing various characteristics of flocks and individual animals, exploration of time trends and comparison of distributions of subgroups may reveal patterns leading to hypotheses about causal relationships.

Registries for dairy and meat sheep are in operation in many countries [11–14]. A main purpose of the registries is the recording of genetic traits that are used in breeding programmes. When mortality records are included, factors associated with lamb loss can be investigated, and studies identifying risk factors and providing heritability estimates for perinatal death based on such registries have been published [13, 14].

Over the past years, there has been a gradual increase in average sheep flock size in Norway and housing conditions and management practices have changed to some extent. The breeding programme run by the Norwegian Sheep and Goat Association and, likely, some changes in feeding regimens have led to an increase in average litter size [15].

The aim of this study was to investigate whether such changes have been accompanied by changes in perinatal survival. Based on data from the Norwegian Sheep Recording System (NSRS), we examined crude perinatal mortality rates in Norway in the period from 2000 through 2010. While perinatal mortality usually has been studied as a single entity, we distinguished between stillbirth and neonatal deaths. Time trends and epidemiological patterns were explored, comparing trends within various geographic locations, and different flock and litter sizes. We also compared variations in prevailing weather conditions and lamb mortality rates. At the flock level, we examined whether there was any relationship between stillbirth and neonatal mortality rates and we also compared losses in two consecutive years.

## Methods

### Characteristics of sheep production in Norway

Sheep in Norway are commonly kept indoors from late in the autumn (October) till late spring; some flocks have voluntary access to a limited outdoor area. Breeding starts in November to December. Thus, lambing takes place in the spring (mainly in April to May), before pasture is available, and occurs indoors or in semi-open sheds under rather close supervision. Ewes and their newborn lambs are usually kept in single pens for a couple of days. They are let out on pasture as soon as weather conditions are satisfactory and are kept in areas close to the farm for a few weeks before they are moved to distant pastures. Usually, ewes and lambs are monitored regularly in the post-parturient period. A description of housing and management of sheep in Norway has been given recently [16].

### Data source and quality control

We used data recorded in the NSRS during the period from 2000 through 2010. The NSRS was established in 1962. Membership is optional. Over the past years, the number of flocks in the NSRS has decreased. However, there has been a proportionally greater decrease in the total number of flocks in the country and the percentage of flocks enrolled in the NSRS has gradually increased from 23 % in 2000 to 27 % in 2010 [15]. Because average flock size has increased to a greater extent in NSRS flocks than in non-member flocks, the percentage of ewes in the NSRS rose from 27 to 42 % during the same time period.

Data on individual animal characteristics and variables related to lambing, weighing, slaughter, disease episodes and death are recorded and reported to the NSRS database. Data were partly reported by local advisors, but an increasing proportion of the data was reported by the farmers themselves, reaching 75 % in 2010 (Mari Langaker, Animalia, personal communication). The main objectives of the NSRS are to provide members with detailed information on performance of their own flock, and to be the basis for the selection of ewes and progeny testing of rams used for breeding. Some of these rams, selected at the national level, are used for artificial insemination, while most of the tested rams are used for tupping in local “ram rings”, which consist of a varying number of flocks. Thus, sheep-owners that have joined the NSRS have direct interest in the quality of the data and will likely be inclined to report complete and correct data.

Data for each lambing of each ewe are stored in one file containing variables for flock identification (composed of numbers for county, municipality within county, flock within municipality), ewe identification (unique number within the flock), date of lambing, lambing ease (normal, moderate assistance, dystocia with prolonged intervention), number of stillborn lambs, and number of liveborn lambs that die before receiving an individual identification number. Observed cases of abortion are also recorded. Liveborn lambs are ear-marked shortly after birth and individual records for all animals with an identification number are stored in another file. Among the variables recorded are flock and animal identification, breed, sex, date of birth, date of slaughter or death for other reasons, dam and sire.

The data sources for our study were the files containing lambing records for the years 2000–2010 and individual animal records for lambing ewes and lambs born in this time period. We extracted for each year all ewes for which a lambing date was recorded. A thorough quality control of the data was carried out. Data from the lambing and individual animal files were examined separately and were also combined and compared for each ewe and flock to assess completeness and consistency of the records.

### Variables included

Mortality rates that were studied were annual proportions of stillborn lambs, liveborn lambs that died between days 0 through 5 after birth (before or after being ear-marked), designated here as early neonatal mortality, and those that died between days 6 through 14 after birth, designated here as late neonatal mortality. In this study, stillborn lambs included lambs that were dead at birth and born close to the expected day of lambing; cases of abortion were not included. At the population level, stillbirth rates and mortality rates for the two neonatal periods were the annual sum of dead lambs within each of the three groups divided by the number of lambs at risk of experiencing the outcome. Thus, the denominators for the three rates were the total number of lambs carried by the dams, the total number of liveborn lambs and the total number of lambs alive at day 6 postpartum, respectively. Annual crude proportions were also compared for subgroups of lambs aggregated within regions, flock sizes and litter sizes and month of birth.

**Fig. 1:**
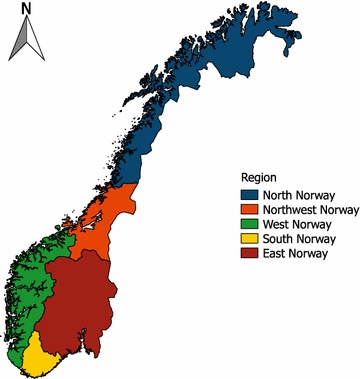
Regions of Norway

A variable identifying region was derived from the flock identification number, which contains the county number. The 19 counties of Norway were grouped into five regions as shown in Fig. 1—North Norway (including Finnmark, Troms and Nordland counties), Northwest Norway (the two counties of Trøndelag), West Norway (Møre og Romsdal, Sogn og Fjordane, Hordaland and Rogaland counties), South Norway (the two counties of Agder) and East Norway (the eight remaining counties). Average flock size increased during the observation period. To compare the trends for both the smallest and largest flocks with those for the medium sized, flock size was divided into four categories (<26 ewes, 26–125, 126–250 and >250) based on the sum of ewes that lambed the particular year. Litter size, which included liveborn and possible stillborn lambs, was grouped into 1, 2, 3, 4 and >4 lambs. The distribution of ewes by some other characteristics was compared for 2000 and 2010. Annual data for the period 2000 through 2010 on average temperature and precipitation in April and May, which are the prime lambing months, were provided by the Norwegian Meteorological Institute. The data were based on gridded datasets that are established by spatial interpolation of monthly precipitation and temperature deviations from a 30 years mean value (1961–1990) according to methods described previously [17–19]. Absolute values were obtained by combining the deviation grids and the grids representing the 30 year mean monthly values. When comparing mortality rates with weather conditions in April and May, only lambs born in April and May were included and mortality records were examined separately for each month.

### Data handling and statistical analysis

Data handling and statistical analysis were performed using SAS (version 9.2, SAS Institute Inc., Cary, NC, USA). Crude time trends were assessed visually by graphing annual mortality rates. The Cochran–Armitage trend test was used to determine whether a significant trend was present across the time period. *P*-values <0.05 (two-sided) were considered significant.

When flock size is small, modest variations in the number of lambs lost can cause relatively large changes in mortality rates. When comparing flock level mortality rates recorded in two consecutive years and stillbirth and neonatal mortality rates recorded within the same flocks, we therefore decided to include only flocks with more than 50 lambing ewes. We used the Pearson correlation coefficient to assess the relationship between rates.

## Results

Table 1 shows the number of ewes initially extracted each year, the numbers excluded for various reasons and the numbers included in the study. In some flocks, several individual animal records were incomplete or missing. Because a non-random recording of animals could not be ruled out, we decided to exclude all ewes in these flocks. For several ewes, particularly in 2009 and 2010, a lambing date had been registered, but no offspring was recorded. In many of these cases, abortion had been recorded and the «date of lambing» was the date of abortion. For the remaining ewes the reason is unknown, but a likely explanation is that, based on the day of breeding, an expected lambing date had been calculated and recorded, whereas the ewe turned out to be non-pregnant. For some ewes, lambing had been recorded twice the same year and for a few ewes there were three records. When repeated records were identical, one was kept in the dataset. When records differed, which could have been due to the recording of an erroneous ewe identity number, none was kept. The final number of lambing ewes included in the study was 3,066,700 and these gave birth to a total of 6,435,715 lambs.

**Table 1:** Number of ewes registered with a lambing date in the Norwegian Sheep Recording System each year from 2000 to 2010, numbers excluded for various reasons, and final numbers of ewes and lambs included in the current study

Year	Exclusions					
	Ewes with a lambing date	Ewes with no lambs^a^	Ewes with >1 record^b^	Ewes in flocks with incomplete records^c^	Ewes in the study	Lambs in the study
2000	281,380	2150	563	2036	276,631	554,007
2001	292,341	2053	1073	1938	287,277	580,445
2002	293,317	3137	865	1576	287,739	587,199
2003	295,714	3150	592	1092	290,880	601,980
2004	290,680	2277	972	1722	285,709	598,821
2005	291,193	3752	582	2337	284,522	598,771
2006	283,146	3311	297	1235	278,303	593,825
2007	272,728	2443	251	1479	268,555	573,721
2008	274,219	3851	215	2065	268,088	575,752
2009	292,088	21,443	124	1636	268,885	582,797
2010	288,536	17,187	154	1084	270,111	588,397

^a^There was a lambing date in the registry, but the ewes were excluded because no lambs (stillborn or liveborn) were recorded

^b^There were two or more records for the same ewe in the same year; one record was kept when records were identical; all were deleted when records were different

^c^All ewes were excluded in flocks with missing lambing records for several ewes

### Characterization of flocks and animals

The number of flocks participating in the NSRS decreased gradually from 4941 flocks in 2000 to 3654 flocks in 2010. Of the 4941 flocks in the NSRS in 2000, 2573 (52.1 %) were still enrolled in 2010, and of the 3654 flocks enrolled in 2010, 1081 (29.6 %) did not participate in 2000.

The average number of lambing ewes per flock increased from 56 in 2000 to 74 in 2010. The distribution of flocks and ewes by flock size category in 2000 and 2010 is shown in Table 2. The proportion of flocks with more than 125 ewes increased. The distribution of the NSRS flocks across the five regions did not change much during the study period; 11–12 % were located in North Norway, 15–16 % in Northwest Norway, 38–40 % in West Norway, 28–29 % in East Norway and 4–5 % in South Norway. The distribution of ewes according to certain characteristics in 2000 and 2010 is shown in Table 3. Average litter size increased from 2.00 in 2000 to 2.19 in 2010. Amongst ewes registered in the NSRS that lambed in 2000, 1.9 % of the 1-year-old ewes and 26 % of the ewes that were 2 years or older gave birth to litters of more than two lambs. In 2010, 6.5 % of the 1-year-old ewes and 38 % of the older ewes gave birth to litters of more than two lambs. In ewes that were 2 years or older, litters of more than 3 lambs were more common in 2010 (5 % of all litters born to ewes in this age group this year) than in 2000 (2 % of the litters). The distribution of the ewes in 2000 and 2010 according to the other variables listed in Table 3 did not differ much. In 2010, 18,855 live-born lambs died within the first 2 weeks of life; 69.5 % died on days 0–2, 13.2 % on days 3–5 and 17.3 % on days 6–14 after birth.

**Table 2:** Distribution of flocks and ewes enrolled in the Norwegian Sheep Recording System in 2000 and 2010 by flock size

	Flock size	2000		2010	
		n	%	n	%
Flocks enrolled	<26	1009	20.4	547	15.0
	26–125	3651	73.9	2602	71.2
	126–250	268	5.4	451	12.3
	>250	13	0.3	54	1.5
Ewes enrolled in flocks	<26	17,044	6.2	8355	3.1
	26–125	214,086	77.4	170,358	63.1
	126–250	41,542	15.0	73,551	27.2
	>250	3959	1.4	17,847	6.6

**Table 3:** Distribution of ewes enrolled in the Norwegian Sheep Recording System in 2000 and 2010 by various characteristics

	2000	2010
	%	%
Litter size		
1	22.9	17.7
2	55.9	51.3
3	19.3	26.8
4	1.7	3.8
>4	0.2	0.4
Age of ewe (year)		
1	21.4	23.1
2	24.0	24.0
3	19.4	18.7
4	15.4	13.7
5	10.3	9.8
6	6.3	6.7
>6	3.2	4.0
Lambing ease		
Normal	78.3	75.0
Assistance, not dystocia	11.0	12.0
Assistance, dystocia	10.7	13.0
Time of lambing		
Prior to April 1	1.1	2.2
April 1–15	10.5	14.0
April 16–30	39.0	40.2
May 1–15	39.3	35.2
May 16–31	9.4	7.9
After May 31	0.7	0.5
Breed		
Norwegian White	77.1	80.4
Spæl	17.6	13.0
Old Norwegian breeds^a^	1.4	2.2
Other^b^	3.9	4.4

Percentage distribution does not include missing values. Missing values: age for 8 ewes in 2000 and 1337 ewes in 2010; lambing ease for 2217 ewes in 2000 and 479 ewes in 2010; breed for 937 ewes in 2010

^a^Includes Furbearing Sheep, Old Norwegian Sheep, Old Spæl

^b^Includes Blazed Sheep, Fuglestad Pied, Grey Trønder, Texel, Cheviot, Oxford Down, Suffolk, Merino, Finnish Landrace, Blackface, Dorset, Charollais, Romney, and various crossbreeds

### Overall trends in stillbirth and neonatal mortality

**Fig. 2:**
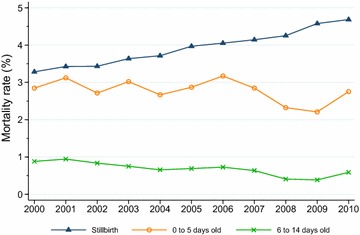
Trends in stillbirth and neonatal mortality rates of live born lambs 0 to 5-days-old and 6 to 14-days-old in flocks enrolled in the Norwegian Sheep Recording System, 2000–2010

Stillbirth rates and neonatal mortality rates from 2000 to 2010 of lambs 0 to 5-days-old and 6 to 14-days-old are displayed in Fig. 2. The proportion of stillborn lambs rose almost linearly from 2000 (3.28 %) to 2010 (4.68 %), an increase of 43 % (*P* < 0.0001, Cochran–Armitage test).

Early neonatal mortality rates varied from year to year (Fig. 2), with a minimum in 2009 (2.21 %) and a maximum in 2006 (3.17 %). However, the Cochran–Armitage test showed that there was a highly significant decreasing trend (*P* < 0.0001) in the early neonatal mortality rates across the entire period from 2000 through 2010. Late neonatal mortality rates exhibited a more evident declining trend (*P* < 0.0001) (Fig. 2), although a marked increase occurred from 2009 to 2010. Minimum and maximum rate was 0.39 % (in 2009) and 0.94 % (in 2001), respectively. Year-to-year variations in early and late neonatal mortality rates tended to be parallel.

### Mortality trends by litter size, age of ewe and breed

**Fig. 3:**
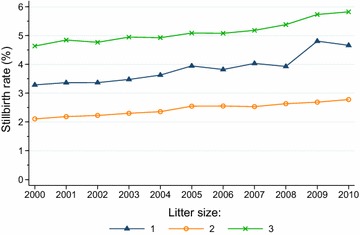
Trends in stillbirth rates for lambs born in litters of 1, 2 and 3 lambs in flocks enrolled in the Norwegian Sheep Recording System, 2000–2010

Figure 3 shows stillbirth rate by litter size. From 2000 to 2010 the proportion of stillborn lambs increased by 42 % (from 3.29 to 4.66 %) in singleton litters, by 32 % (from 2.11 to 2.78 %) in twin litters and by 26 % (from 4.64 to 5.83 %) in triplet litters (*P* < 0.0001, Cochran–Armitage test). Also among lambs in quadruplet litters there was a significant increasing trend (*P* < 0.0001) but the proportion of stillborn lambs fluctuated somewhat from year to year (minimum 8.56 % in 2002, maximum 9.94 % in 2010). Stillbirth rates in 1-year-old and >1-year-old ewes increased in a parallel manner during the study period, from 2.04 and 2.51 % in 2000 to 3.41 and 3.58 % in 2010, respectively; the trends for both groups were highly significant (*P* < 0.0001). For ewes of the Norwegian White breed stillbirth rates rose from 2.47 % in 2000 to 3.66 % in 2010, while for the Spæl breed the stillbirth rates rose from 2.33 to 3.12 %; the trends for both breeds were highly significant (*P* < 0.0001).

**Fig. 4:**
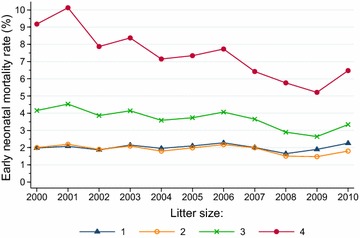
Trends in neonatal mortality rates of 0 to 5-days-old lambs born in litters of 1, 2, 3 and 4 lambs in flocks enrolled in the Norwegian Sheep Recording System, 2000–2010

**Fig. 5:**
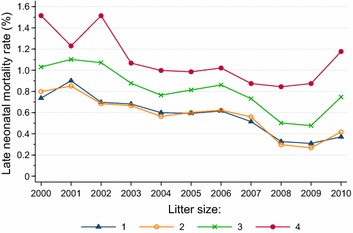
Trends in neonatal mortality rates of 6 to 14-days-old lambs born in litters of 1, 2, 3 and 4 lambs in flocks enrolled in the Norwegian Sheep Recording System, 2000–2010

The annual fluctuations in early (Fig. 4) and late (Fig. 5) neonatal mortality rates displayed a similar pattern within the various litter sizes. Whereas early neonatal mortality did not show any significant trend across the study period for singleton lambs, there was a significant declining trend (*P* < 0.0001) for twins, triplets and quadruplets. Early neonatal mortality rates were invariably higher among triplet lambs (between 2.64 and 4.52 %) and quadruplets (between 5.52 and 10.12 %) than among singleton lambs (between 1.65 and 2.28 %) and twins (between 1.47 and 2.19 %). Late neonatal mortality showed a significant (*P* < 0.0001) declining trend within all litter sizes, with rates varying from 0.31 to 0.90 % in singleton lambs, from 0.27 to 0.85 % in twins, from 0.48 to 1.10 % in triplets and from 0.84 to 1.52 % in quadruplets.

### Mortality trends and weather conditions

**Fig. 6:**
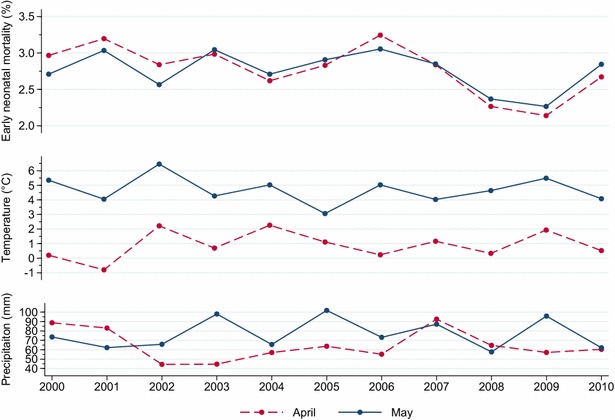
Trends in neonatal mortality rates of 0 to 5-days-old lambs born in April and May 2000–2010 in flocks enrolled in the Norwegian Sheep Recording System (*upper*). National average temperature (*middle*) and national average precipitation (*lower*) in April and May 2000–2010

**Fig. 7:**
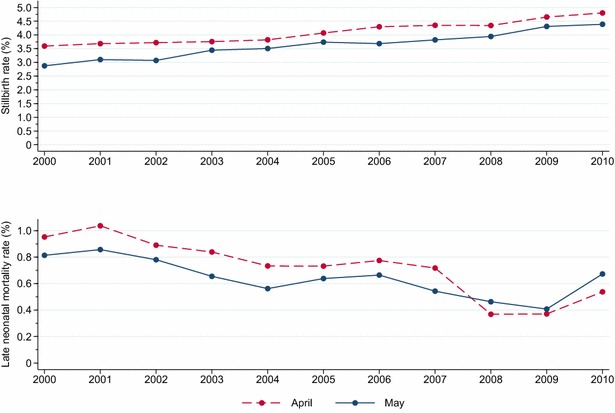
Trends in stillbirth rates (*upper*) and neonatal mortality rates (*lower*) of 6 to 14-days-old lambs born in April and May 2000–2010 in flocks enrolled in the Norwegian Recording System

In the study period, national average temperatures in April varied from −0.8 to 2.3 °C and were each year lower than average temperatures in May (minimum 3.1 °C, maximum 6.5 °C) (Fig. 6). Year-to-year temperature variations for these 2 months were similar. With the exception of May 2006, there was a clear tendency that early neonatal mortality in April and May was relatively high in years when average monthly temperature was relatively low (Fig. 6); however, there was no systematic difference in early neonatal mortality rates between April and May. Average precipitation was most years higher in May (minimum 57 mm, maximum 101 mm) than in April (minimum 44 mm, maximum 94 mm) (Fig. 6). There was no clear relation between early neonatal mortality and average precipitation. Stillbirth and late neonatal mortality did not show any obvious relationship with temperature or precipitation. The stillbirth trends across the study period were similar for April and May, but the proportion of stillborn lambs was constantly higher in April (Fig. 7). The greatest difference was in 2000 when the stillbirth rate was 25.0 % higher in April than in May (3.58 versus 2.86 %); the smallest difference was in 2009 when the rate was 8.0 % higher in April (4.65 versus 4.31 %). In most of the study period, late neonatal mortality rates were higher for lambs born in April than for lambs born in May (Fig. 7).

### Mortality trends by flock size

**Fig. 8:**
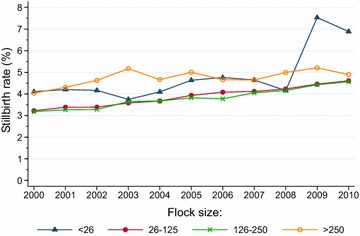
Trends in stillbirth rates in flocks with <26, 26–125, 126–250 and >250 lambing ewes registered in the Norwegian Sheep Recording System, 2000–2010

An increasing stillbirth rate was observed within all flock size categories from 2000 to 2010 (*P* < 0.0001, Cochran–Armitage test) (Fig. 8). In flocks with 26–250 ewes, which during the study period included 90–92 % of all ewes enrolled in the NSRS, there was an almost linear increase in the average stillbirth rate from 3.20 % in 2000 to 4.58 % in 2010. Flocks with <26 or >250 lambing ewes had higher average stillbirth rates than the medium sized flocks throughout the study period. In the smallest flocks, stillbirth rates varied, and a substantial increase occurred from 2008 (4.16 %) to 2009 (7.53 %); a high rate was present also in 2010 (6.89 %).

**Fig. 9:**
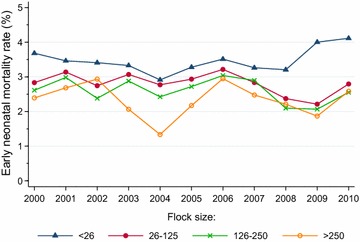
Trends in neonatal mortality rates of 0 to 5-days-old lambs in flocks with <26, 26–125, 126–250 and >250 lambing ewes registered in the Norwegian Sheep Recording System, 2000–2010

Early neonatal mortality rates within flock size categories are displayed in Fig. 9. Similar patterns of year-to-year variation were observed for flocks with 26–125 ewes and 126–250 ewes. In flocks with less than 26 ewes, the early neonatal mortality rate (minimum 2.91 %, maximum 4.11 %) was higher than in the larger flocks throughout the study period. Flocks with >250 lambing ewes had most years lower rates than smaller flocks. The Cochran–Armitage test showed highly significant declining trends for the flock size categories 26–125 and 126–250 (*P* < 0.0001), but no significant trend for the largest and smallest size categories.

**Fig. 10:**
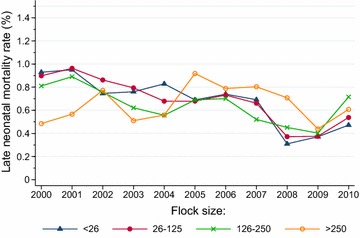
Trends in neonatal mortality rates of 6 to 14-days-old lambs in flocks with <26, 26–125, 126–250 and >250 lambing ewes registered in the Norwegian Sheep Recording System, 2000–2010

Late neonatal mortality did not show any significant trend in flocks with >250 lambing ewes, but there was a highly significant declining trend (*P* < 0.0001) within the other flock size categories (Fig. 10). No systematic differences related to flock size were detected.

### Regional mortality trends

Stillbirth rates increased within all regions of Norway and no substantial regional differences were found. Early and late neonatal mortality rates showed year-to-year variations similar to those found for the total population. Except for 2002, early neonatal mortality was each year lower in West Norway than in all the other regions. South and East Norway clearly tended to have the highest early neonatal mortality rates. There was a highly significant declining trend in early neonatal mortality rates (*P* < 0.0001) in all regions except for South Norway. From 2000 through 2007, late neonatal mortality rates were lowest in North Norway, but there was no significant trend across the entire study period in this region. In each of the other regions, there was a highly significant downward trend in late neonatal mortality rates (*P* < 0.0001).

### Flock level variations and correlations

In 2010, 2153 flocks had more than 50 lambing ewes. Mean (standard deviation) of stillbirth and early and late neonatal mortality rates was 4.48 (3.00), 2.65 (3.17) and 0.57 (0.85), respectively. The within-flock correlation between stillbirth and early neonatal mortality rates and between early and late neonatal mortality rates were r = 0.13 (*P* < 0.0001) and r = 0.18 (*P* < 0.0001), respectively. A total of 1928 flocks had more than 50 lambing ewes in both 2009 and 2010. Stillbirth rates and early neonatal mortality rates recorded in these flocks in 2009 were strongly correlated with those recorded the subsequent year, r = 0.43 (*P* < 0.0001) and r = 0.40 (*P* < 0.0001), respectively. Also for late neonatal mortality rates, the correlation between rates in 2009 and 2010 was highly significant, r = 0.23 (*P* < 0.0001).

## Discussion

This study, which comprised a large proportion of the sheep population in Norway, revealed a markedly increasing trend in stillbirth rate from 2000 through 2010. The trend was remarkably linear, suggesting a gradually increasing impact of major risk factors for stillbirth at the population level. At the end of the study period, 4.7 % of all lambs born were stillborn, which are close to rates reported from other countries. A study performed in the UK reported 4 % stillborn [20] and a long-term study of Merino sheep in South Africa found a stillbirth rate of 4.3 % [21]. Two studies in Scottish Blackface flocks in the UK found, respectively, that 5.3 and 8.0 % of the lambs were stillborn [4, 22]; however, these studies also included lambs that died during the first day of life in their definition of stillbirth.

Multiple pregnancy increases the risk of foetal death in sheep [23, 24]. Average litter size of ewes in the NSRS increased continuously in the study period, which could be expected to be a major driving force behind the stillbirth trend. We indeed found that the stillbirth rate was consistently higher among lambs in triplet litters than among singleton and twins, and even higher among quadruplets. However, the steady rising trend was present within each of the litter size subgroups, clearly indicating that other risk factors were involved. The change was most pronounced in single lamb litters, in which the stillbirth rate increased by more than 40 % in the study period. Lambing difficulties are common in many sheep breeds [25, 26], including the Norwegian White, which is the predominant breed in Norway. Large single lambs are an important cause of dystocia [27] particularly in 1-year-old ewes, and one might consider whether increased occurrence of lambing difficulties was a factor contributing to the rising trend. The proportion of ewes with dystocia was somewhat higher in 2010 than in 2000; however, the increase was small and proportionately less in ewes with single lambs than in those with multiple litters. Trends in stillbirth rate for 1-year-old and >1-year old ewes were parallel. Similar trends were observed in stillbirth rates of the two major breeds. Thus, age and breed distributions were apparently not factors contributing to the increase in stillbirth rates.

Early neonatal mortality varied from year to year, with crude rates around 3 %. A modest declining trend was present when considering the entire study period, which was due to reduced loss in the final years. Greater neonatal loss of lambs has been recorded in other countries. Two studies reporting neonatal mortality rates within 3 and 4 days of age, respectively, found that 9.4 and 9.0 % of all liveborn lambs were lost [28, 29]. A third study reported a 3.0 % loss during the first day of life and an additional loss of 2.7 % over an undefined postnatal period [20]. Neonatal mortality rates in liveborn lambs up to 7 days of age of 12.1 and 14.2 % have been reported from UK and New Zealand, respectively [3, 28]. In accordance with other studies [20, 28, 30], we found that a main proportion of neonatal deaths occurred very early. Seventy percent of the lambs that died within 2 weeks of age died during the first 2 days, demonstrating that events related to lambing and the immediate post-lambing period are critical for lamb survival.

Several studies have shown that in flocks managed permanently outdoors, harsher conditions in terms of low temperatures, wind and precipitation decrease early neonatal lamb survival [10, 29, 31]. With very few exceptions, lambing takes place indoors in flocks in Norway, which should be expected to prevent neonatal deaths caused by adverse weather conditions. However, there was a tendency that in years when early neonatal loss peaked, outdoor temperatures during the months of lambing were low, suggesting that weather could perhaps affect lamb survival. A recent survey of sheep enterprises in Norway found that almost 40 % of sheep barns were uninsulated [16] and some semi-open buildings do exist. In uninsulated barns, indoor temperatures correlate with outdoor temperatures [32] and lamb viability might be reduced in cold weather.

In agreement with previous studies [3, 29], we found that neonatal mortality rates varied considerably with litter size. Whereas Hatcher et al. [3] recorded a higher mortality rate among twins than among singletons during the first week of life, we found almost equal annual rates for twins and singletons in most of the study period and in the last 2 years somewhat higher rates occurred in singletons. For larger litters, mortality rates were much higher. However, a clear declining trend was observed for triplets and even more for quadruplets, which might suggest that management routines for taking care of lambs in multiple litters have improved. Artificial rearing of surplus lambs may have become more widespread during these years; however, data on this issue are lacking. In the breeding program for both Norwegian White and Spæl, main emphasis is put on ewes’ productivity as determined by live animal or slaughter weights of progeny [33], which might have led to better mothering abilities. In a recent survey, over 80 % of the farmers responded that at least some ewes in their flock reared triplets (Holmøy et al., unpublished). The decreasing neonatal mortality rates between 2006 and 2009 might have been due partly to an active information campaign by the Norwegian Sheep Health Service in this period, encouraging sheep farmers to ensure adequate intake of colostrum by newborn lambs.

Late neonatal mortality rates were lower than 1 % throughout the study period. A moderate declining trend and a modest year-to-year variation were present when considering the entire period. Binns et al. [20] reported that 4.0 % of total postnatal deaths occurred in lambs more than 7 days old. Mortality rates in lambs 7–30 days old were 2.2 and 3.1 % in a Scottish Blackface flock and an Australian Merino flock, respectively [3, 4].

Large differences in stillbirth and neonatal mortality rates between flocks and rather strong correlation between rates in two consecutive years within the flocks clearly suggest that flock level risk factors are important and to some extent stable from year to year within a flock. Similar findings have been reported previously [20]. One might suspect that rates would vary between the separate regions of Norway due to systematic differences in environmental conditions; however, differences were small and time trends quite similar. Average number of lambing ewes per flock increased during the study period and management and individual animal monitoring may differ according to flock size. In most of the period, the largest flocks experienced somewhat greater stillbirth rates than the medium-sized; however, the difference declined with time and the upward trend of the medium-sized flocks was of the same magnitude as that of the entire population.

Factors that likely vary between flocks and affect the risk of perinatal mortality are how well lambing is monitored, whether appropriate birth assistance is provided when needed, and whether colostrum intake of newborn lambs is ensured [16]. One might expect that managing a small flock facilitates individual monitoring of ewes and newborn lambs during the lambing season. However, the smallest flocks experienced rather high stillbirth rates and the highest early neonatal losses throughout the study period. The smallest flocks are likely to be owned by farmers having sheep as their secondary occupation, which may imply reduced monitoring.

Most studies of perinatal lamb mortality do not distinguish between stillbirths and early neonatal deaths but consider these as a common entity when, for instance, evaluating determinants for perinatal deaths. We found that correlation at the flock level between stillbirth and early neonatal mortality rates was relatively low. A natural interpretation is that, although stillbirths and early neonatal deaths may share some common risk factors, major parts of the causal complexes are different, suggesting that analysis of risk factors should be carried out separately for late foetal and early neonatal deaths.

A strength of the present study was that it was based on a national sheep registry where a large proportion of ewes in Norway is enrolled. Flocks in the registry were distributed across all counties of Norway and the distribution of members within each county corresponded well to the overall distribution of sheep production units in Norway [34]. Routines for the recording of events in NSRS flocks did not change much during the study period. A large number of animals were included in the study and a thorough quality control of the data was carried out. We therefore consider the data reliable and the trends observed across this time period to reflect real changes in the sheep population in Norway fairly well. Participation of flocks in the NSRS was dynamic; just above half of the flocks enrolled in 2000 were still enrolled in 2010 and almost a third of those enrolled in 2010 had joined the NSRS after 2000. Temporal trends presented were therefore based on crude population level data. Trends within levels of certain variables exhibited patterns that suggested causal relationships with stillbirth and neonatal mortality. However, inferences regarding relations at the individual animal level generally cannot be based on patterns at the population level and suggestive findings should be verified in studies with appropriate design.

## Conclusion

We found a substantial increase in ovine stillbirth rate in recent years in Norway. In the same time period, there has been a corresponding increase in litters of three or more lambs, in which the stillbirth rate was higher than in litters of singletons and twins. However, the stillbirth rate increased over time within each litter size, indicating that gradual changes over time in other, yet unidentified, factors operating at the population level apparently have contributed to the overall increased stillbirth rate. Despite the increase in litters of more than two lambs, crude rates for early and late neonatal mortality displayed moderate declining trends. Early neonatal mortality rate exhibited year-to-year variations, which partly might be explained by variations in outdoor temperature in the lambing season.
